# Toward a more holistic method of genome assembly assessment

**DOI:** 10.1186/s12859-020-3382-4

**Published:** 2020-07-06

**Authors:** Adam Thrash, Federico Hoffmann, Andy Perkins

**Affiliations:** 1 Institute for Genomics, Biocomputing & Biotechnology, Mississippi State University, Mississippi State, MS USA; 2 Department of Computer Science and Engineering, Mississippi State University, Mississippi State, MS USA; 3 Department of Biochemistry, Molecular Biology, Entomology and Plant Pathology, Mississippi State University, Mississippi State, MS USA

**Keywords:** Genome assembly, Contiguity, Completeness, Correctness, N50

## Abstract

**Background:**

A key use of high throughput sequencing technology is the sequencing and assembly of full genome sequences. These genome assemblies are commonly assessed using statistics relating to contiguity of the assembly. Measures of contiguity are not strongly correlated with information about the biological completion or correctness of the assembly, and a commonly reported metric, N50, can be misleading. Over the years, multiple research groups have rejected the overuse of N50 and sought to develop more informative metrics.

**Results:**

This paper presents a review of problems that arise from relying solely on contiguity as a measure of genome assembly quality as well as current alternative methods. Alternative methods are compared on the basis of how informative they are about the biological quality of the assembly and how easy they are to use. A comprehensive method for using multiple metrics of measuring assembly quality is presented.

**Conclusions:**

This study aims to report on the status of assembly assessment methods and compare them, as well as to offer a comprehensive method that incorporates multiple facets of quality assessment. Weaknesses and strengths of varying methods are presented and explained, with recommendations based on speed of analysis and user friendliness.

## Background

Genome assembly is the process of assembling biological reads from sequencing into larger sequences called *contigs*. A common method of quantifying the quality of genome assemblies is to compute statistics about contiguity. For example, the assembly for *M. musculus* at the National Center for Biotechnology Information (NCBI) website reports features like N50 (a number which represents the smallest contig such that half the genome is represented by contigs of size N50 or larger [1, 2]), total sequence length, gaps between scaffolds, and number of contigs [3]. However, there is no obvious relationship between these numbers and whether a genome contains any useful information. Indeed, many papers published in the past ten years have cited this lack of a relationship, in addition to N50’s own issues, as a reason to replace or supplement N50.

The final output of the assembly process may contain errors; these errors may arise as a result of problems that occurred in the sequencing or problems that occurred during the assembly process. These errors can be discussed in terms of contiguity, completeness, and correctness. These three features, sometimes called the three Cs, are defined below. An ideal genome is contiguous, complete, and correct.

Contiguity is related to the size and number of contigs. If a goal of assembly is to reflect the contiguity of the genome in vivo, then the assembly process seeks to maximize the size of the contigs and to minimize the number of contigs to reflect the true number and size of the chromosomes in the organism. Contiguity errors may arise due to assembler parameters that allow unrelated contigs to be joined or that prevent related contigs from being joined.

Completeness is determined by the content of contigs, especially with regard to gene content. A contiguous genome that contains no gene content is not useful for downstream analysis. Completeness errors can arise in sequencing (important genes may not be sequenced) or they may arise in the assembly process (genes may end up in discarded contigs).

Correctness is concerned with the ordering and location of contigs. A correct genome assembly has the same order as the true genome. If contigs are incorrect, they may have inversions, relocations, or translocations with respect to the true genome [1].

A discussion of types of assembly errors is not a discussion of three separate facets of assembly assessment. Contiguity errors can be discussed in terms of how those errors are misjoins in relation to the true genome. Some measures of completeness are related to contiguity. For example, fragmentation of the genome (a measure of contiguity) is related to fragmentation of the genes [4]. An incorrectly collapsed heterozygous allele in an assembly contributes to inaccurate contiguity statistics.

In the last decade, multiple papers have discussed genome assembly assessment. Many of these papers begin with an assertion that contiguity or N50 specifically is not well-correlated with genome correctness or completeness. Alternatives to N50 are then presented; these alternatives are either meant to supplement or replace N50.

Salzberg et al. discuss comparisons of assemblies and assemblers in their paper. They present genome assemblies from many assemblers and discuss which genome assemblies are better. In their paper, they compare genomes using measures of contiguity like N50 and number of contigs, in addition to a metric called e-size that they developed. They note that genome correctness is not well-correlated with statistics about contiguity. They also note that N50 can be misleading; in the case of misjoins, contigs are larger than they should be, which inflates the N50 score. To address this issue, Salzberg et al. developed e-size, which measures the size of a contig containing a randomly selected base on the genome. The paper states that this metric can help answer questions about how many genes are completely contained in contigs or scaffolds rather than fragmented. While e-size is more robust than N50, e-size is still affected by misjoins. Because it is a continuous measure rather than a discrete measure, the effect is less noticeable [1].

Meader et al. developed a method of genome assembly assessment that relies on alignments to genomes of closely related species. In their paper, they assert that N50 measures the assembler’s ability to combine reads in large seamless blocks but does not reflect fine-scale inaccuracies like substitutions, insertions, or deletions. While their method is rooted in biology, its requirement of a reference genome makes it much less useful for those who are assembling genomes of species without good references [5].

Utturkar et al., in their study of hybrid assembly techniques, write that N50 and the number of contigs are widely used but don’t always correlate with assembly quality. Instead, they relied on CGAL and REAPR, two tools that will be discussed later [6].

Seemann et al. use many features for their comparison of animal genomes: nucleic acid conservation of highly conserved protein-coding and ultraconserved elements (UCs), amino acid homology of universal single-copy orthologs, structure conservation of housekeeping RNAs, assembly sequence quality, and assembly contiguity. They calculate the Euclidean distance of the first three principle components of each species and human, which they use as a gold standard of assembly [7].

Hunter et al. developed a tool called REAPR [8] to address their issues with N50. Notably, they assert that N50 only indicates contiguity of the genome, not accuracy, and that it is often boosted by improperly joined contigs. Their approach is to correct N50 by using data from the reads used in assembly to discover misjoins and break them apart to produce a more correct assembly with a lower N50. While this tool performs and important function, its output still reflects a desire to use contiguity as a measure of genome assembly quality.

Rahman and Pachter developed a method that computes the likelihood of an assembly given the reads that were used to create the assembly [9]. Rahman and Pachter created a generative model for sequencing that requires an alignment of the reads to the assembly, which they then use to determine the likelihood of those reads being from the assembly according to their model. CGAL provides no explicit information about gene content or correctness to its users. CGAL does not require a reference genome, which is helpful for researchers that don’t have a reference for their species.

Simão et al. describe N50 as a technical measure that does not reflect gene content, and developed a tool called BUSCO, which searches for benchmarking universal single-copy orthologs (BUSCOs) in an assembly. BUSCO measures these orthologs by counting complete single-copy BUSCOs, fragmented BUSCOs, missing BUSCOs, and duplicate BUSCOs. The authors of BUSCO note that duplicate BUSCOs may represent misassemblies where a heterozygous allele failed to collapse into the assembly properly and was retained as a contig [10].

Gurevich et al. developed a tool for comparing assemblers and assemblies called QUAST. QUAST works with or without a reference genome and introduces metrics related to an assembly’s alignment to a reference in order to counter the possibility of an artificially inflated N50. QUAST measures several factors related to contiguity, structural elements, and functional elements and provides the most information about an assembly when used with an annotated reference genome. Without a reference genome, QUAST uses gene prediction to report the number of predicted genes [11].

Thomas and Hahn developed a tool called Referee. While they make no claims about N50 in their paper, they stress the importance of correct bases in a genome assembly because those errors effect downstream analysis. Referee uses the quality information from the reads used in an assembly to determine the likelihood of having a specific base at every position in the genome and can correct the genome if another base is more likely. Referee does not address correctness directly in terms of misjoins, translocations, or relocations, nor does it report a single metric to the user (it reports the likelihoods of every base in the genome instead). However, Referee serves an important function in ensuring that the best base from all reads that overlap a position is selected [12].

Most of these papers found that contiguity, specifically N50 alone, was insufficient as the sole metric of genome assembly quality. These papers provide supplemental metrics, corrective metrics, or entirely alternative metrics to supplement what they saw as a deficient metric for assessing assembly quality.

Before any discussion of these metrics and their alternatives, an exploration of the goal of genome assembly is helpful. As stated in the introduction, an ideal genome scores well in all three categories of contiguity, completeness, and correctness. For some projects, the goal of genome assembly is improving the assembly for others to use. For others, genome assembly is a means to an end, such as analysis of RNA-seq data. In both cases, there is an expectation that a genome assembly will be used for some downstream analysis. In order to best perform this downstream analysis, researchers need to be certain of the quality of their assembly.

## Methods

To explore genome assembly assessment methods with real data, 800 vertebrate genomes were downloaded from NCBI and assessed with abyss-fac (included with ABySS v2.0.1)[13] and BUSCO (v3) [10, 14]. These genomes represent all of the vertebrate genomes that were available from NCBI at the time in mid-2018. Of these, 797 were successfully assessed. Abyss-fac and BUSCO are publicly available and do not require the reads used to make the assemblies as input (unlike REAPR and CGAL, for example). Seven features were selected and computed to describe each genome. N50, e-size, and number of contigs were selected for contiguity; complete single-copy BUSCOs, fragmented BUSCOs, and missing BUSCOs were selected to describe completeness; and duplicate BUSCOs were selected to describe correctness, since duplicate BUSCOs may arise from the failure of an assembler to correctly assemble heterozygous alleles. These features and the scripts used in this analysis are available in the GitHub repository provided at the end of this paper.

**Fig. 1:**
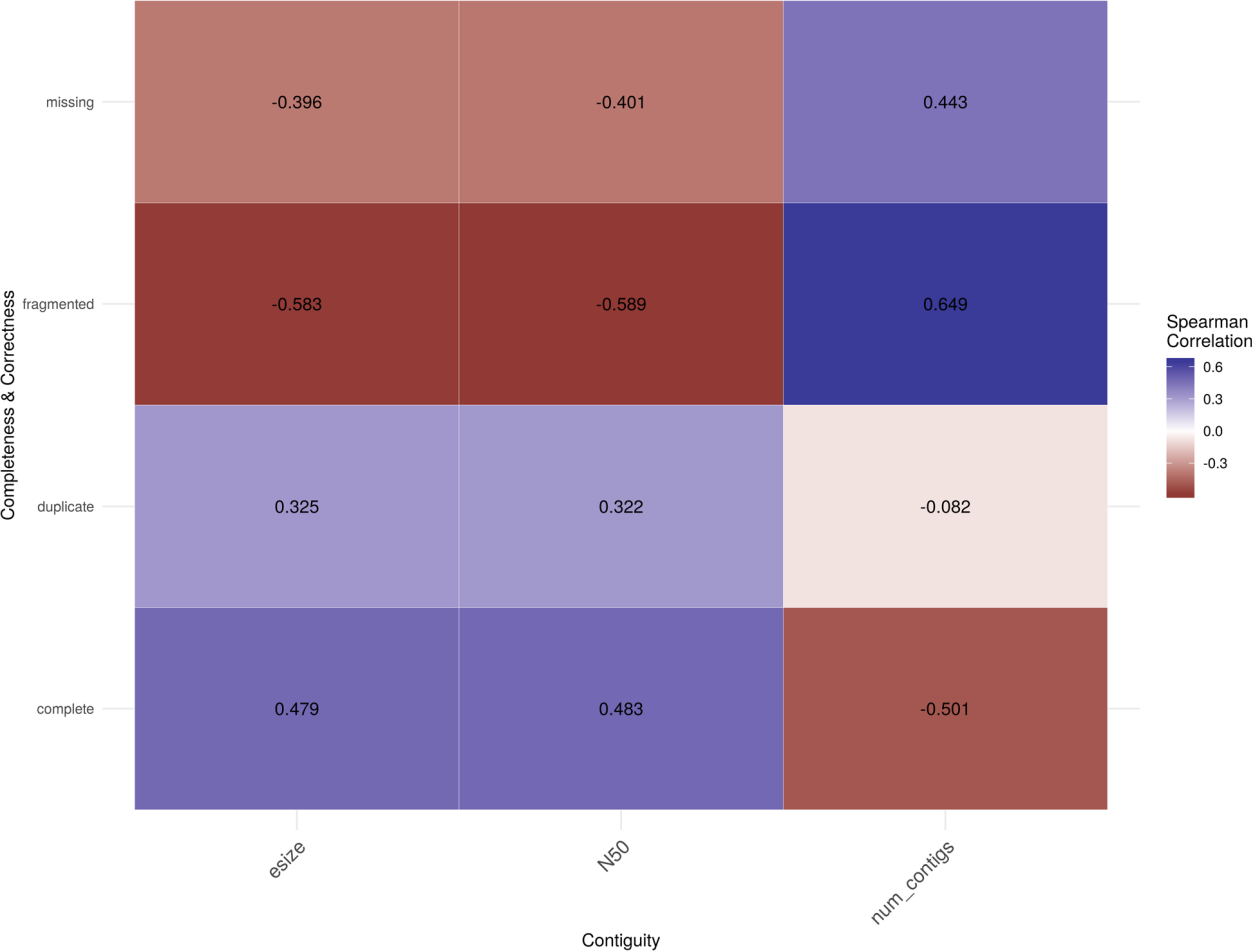
Correlation of contiguity and completeness/correctness

Correlation testing using Spearman’s rank-order correlation was performed to validate the claims that measures of contiguity and measures of completeness are not well-correlated. Statistically insignificant correlations (*p*>0.05) were removed and correlations among the same facet (contiguity/contiguity, for example) were also removed. The resulting image (Fig. 1) shows the correlations discovered, the direction of the correlation, and the strength of the correlation.

In an attempt to make genome assembly quality assessment quick, simple, and intuitive, a metric was developed using information from the vertebrate data set. The number of contigs was selected to present contiguity for two reasons. First, it has a slightly better correlation to the BUSCO features as shown in Fig. 1. Second, the number of contigs has a roughly known target in vertebrates. The red king crab (*Paralithodes camtschaticus*) has the maximum number of chromosomes in animals at 208 chromosomes, so we can use this number as an upper bound on the number of desired contigs in our assemblies.

**Fig. 2:**
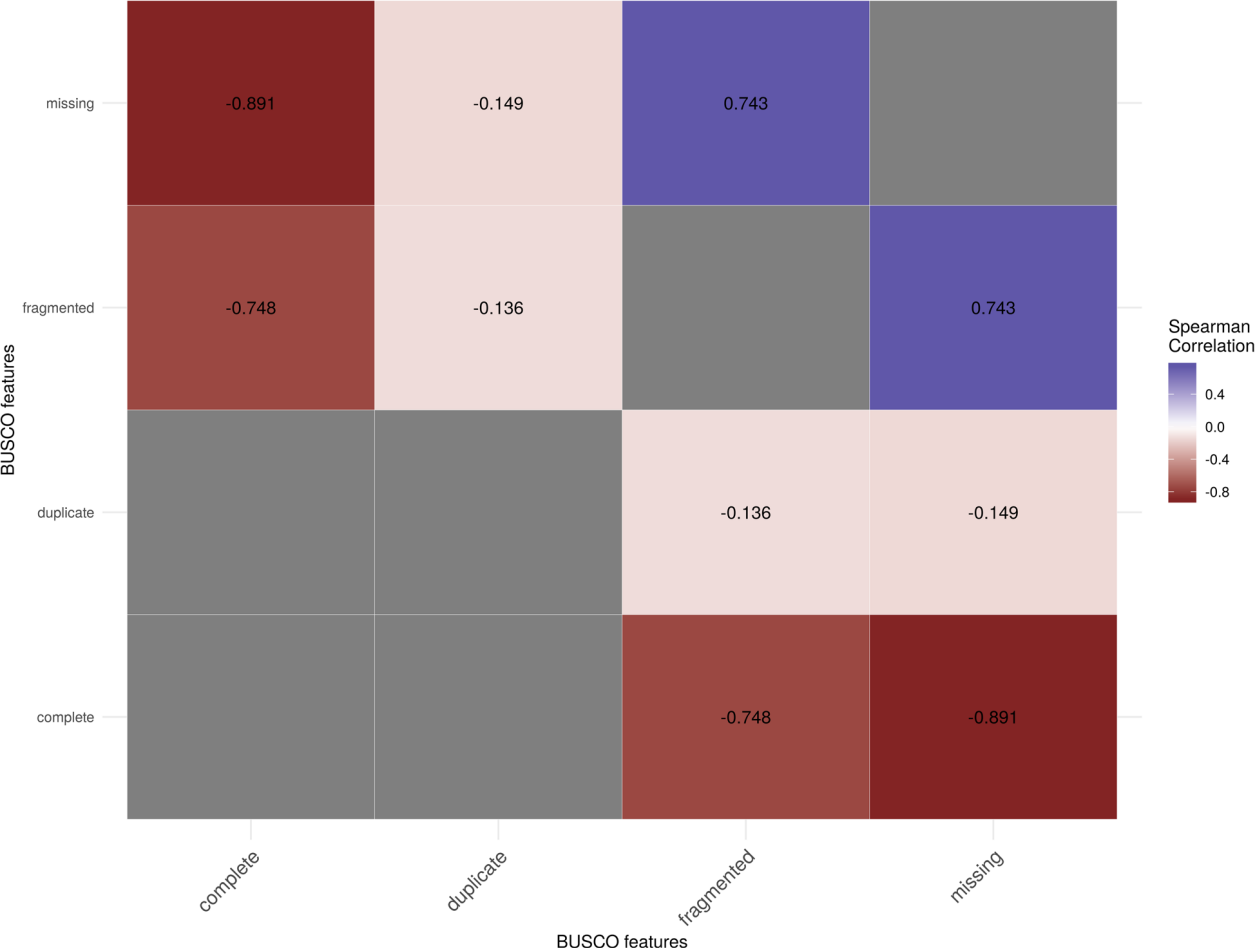
Correlation of BUSCO (benchmarking universal single-copy orthologs) statistics

In order to select representative features of completeness from BUSCO, the correlation between BUSCO’s features was calculated and plotted as shown in Fig. 2. The percentage of complete single-copy BUSCOs was selected to represent completeness. Both the percentage of fragmented single-copy BUSCOs and the percentage of missing single-copy BUSCOs are strongly correlated with the percentage of complete single-copy BUSCOs; therefore, the percentage of complete single-copy BUSCOs was selected to represent all three features.

The percentage of duplicate BUSCOs was not well-correlated with any other BUSCO features or any features representing contiguity (Spearman’s *ρ*=−0.009,*p*=0.808). It was selected as the representative of correctness.

The metric computed involves a determination of an “ideal” genome for the particular assembly in question. In this definition of an ideal genome, all or nearly all single-copy BUSCOs are captured completely, duplicate BUSCOs are low or non-existent, and the number of contigs is equal to the expected number in the species or a closely related species. These three factors allow the computation of a Euclidian distance from the current genome to the ideal genome. This metric does assume that a researcher has some knowledge of the expected number of chromosomes. Eq. 1 shows how this metric is computed, where *i*_*c*_ is the ideal number of complete single-copy BUSCOs, *b*_*c*_ is the predicted number of single-copy BUSCOs, *i*_*d*_ is the the ideal number of duplicate BUSCOs, *b*_*d*_ is the predicted number of duplicate BUSCOs, *n*_*c*_ is the number of chromosomes, and *n* is the number of contigs. The comparison between number of contigs versus number of chromosomes is converted to a percent, since the number of contigs can be very high. By converting the relationship between number of chromosomes and number of contigs to a percentage, all three comparisons are on the same scale of 0-100.

1 $$  d_{i} = \sqrt{(i_{c} - b_{c})^{2} + (i_{d}-b_{d})^{2} + (100-(\frac{n_{c}}{n})*100)^{2}}  $$

## Results and discussion

Some of the features of contiguity appeared to be moderately correlated with features of completeness, while others were less strongly correlated. The percentage of fragmented BUSCOs has a moderate correlation to the three factors of contiguity in the comparison. This relationship can be explained by considering the the relationship between contiguity and fragmented BUSCOs — as the number of contigs decreases, any BUSCOs that might be fragmented across contigs are instead now joined. Some relationship between contiguity and fragmented genes is expected and therefore not surprising when fully considered [4].

**Fig. 3:**
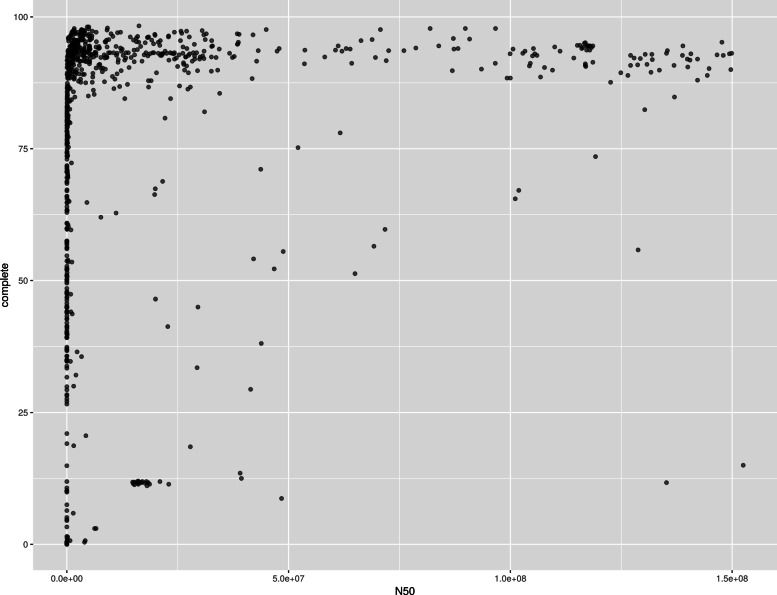
N50 (a number which represents the smallest contig such that half the genome is represented by contigs of size N50 or larger [1, 2]) vs Percentage of Complete Single-Copy BUSCOs (benchmarking universal single-copy orthologs)

Complete single-copy BUSCOs were somewhat correlated with measures of contiguity. In order to explain this correlation, N50 and the percentage of complete single-copy BUSCOs were plotted against each other. N50 was selected because it is the metric that the authors of the reviewed papers critiqued. The initial plot had two outliers with N50 > 2e8 that heavily affected the scale of the plot; these outliers were dropped for the sake of a clearer visualization as seen in Fig. 3.

Deciphering a relationship between N50 and the percentage of complete single-copy BUSCOs is difficult based on the information provided in this plot. There are many genomes with high completeness plotted for a large range of N50 values from high to low. There are also many genomes with low N50 that have a percentage of complete single-copy BUSCOs ranging from 0 - 100.

In other words, given a low N50, no prediction about the percentage of complete single-copy BUSCOs can be made. Given a high N50, a high percentage of complete single-copy BUSCOs seems more likely but is not guaranteed. The inverse is also true — given a low percentage of complete single-copy BUSCOs, the N50 is likely to also be low. Given a high percent complete, N50 can range from 0 to 2e8. The quality of the genomes uploaded to NCBI becomes apparent as well.

The percentage of missing BUSCOs and the percentage of complete single-copy BUSCOs is similar, although the correlation is a negative correlation for the percentage of missing BUSCOs. While this relationship to N50 was plotted, the plot was roughly similar to the plot in Fig. 3 after an inversion along the y-axis and thus is not presented. This result is expected; in most cases, when the percentage of complete single-copy BUSCOs increases, the percentage of fragmented single-copy BUSCOs decreases (Spearman’s *ρ*=−0.8906630).

Duplicate BUSCOs were not well correlated with any measure of contiguity.

Of all the alternative methods of measuring genome assembly quality presented, few of them present information about all three facets of genome assessment in a clear, concise manner. Some alternatives provide metrics that still do not provide information about all facets; other alternatives present so many metrics that gaining a clear understanding of all three facets of a genome assembly can be difficult. Still other methods use some sort of gold-standard.

With the presented distance metric, genome researchers encode their domain knowledge into the formulation of an ideal genome. If they have some knowledge that their species’s genome has a high percent of duplicated genes, they can encode this knowledge into their ideal genome. In this way, the metric encodes human subject expert knowledge and is a comparison to an ideal version of itself rather than to another species’s genome. A perfect score is zero or close to zero, and high scores represent genomes with a flaw in one or more of the facets. Additionally, this metric is extensible; new terms can be added to the equation so long as those terms can be scaled to run from 0 - 100. For example, CGAL’s likelihood scores could be added to this distance metric.

In Table 1, the distances were computed to the ideal genome for a selection of genomes from the vertebrate dataset. In each case, the number of desired chromosomes was taken from the Animal Genome Size Database [15]. Not all of the genomes in this database reported number of chromosomes, but the distance metric was calculated for 316 of the 797 genomes in the vertebrate dataset. The ideal percent of complete single-copy BUSCOs was set to 100%, and the ideal percent of duplicate BUSCOs was set to 0%. However, these distances inherently reflect the bias of the author in how the ideal genome for each of these assemblies should be represented. Additionally, these distances are presented as an example of the types of features one might expect to see associated with particular distances, rather than any attempt to assign a ranking to these genomes.

**Table 1:** The top 5 genome assemblies with the smallest distance, 20 randomly selected genome assemblies, and the bottom 5 genome assemblies with the largest distance

Species	N50	num_contigs	complete	duplicate	distance
*Oryzias latipes*	31200000	24	95.70	1.00	4.41
*Oryzias latipes*	32840000	24	94.50	1.00	5.59
*Oryzias latipes*	28850000	24	92.00	1.10	8.08
*Macaca fascicularis*	133600000	21	89.90	1.00	10.15
*Homo sapiens*	149700000	24	90.00	1.10	10.89
*Falco peregrinus*	88200000	34	94.00	0.30	27.14
*Seriola quinqueradiata*	5609355	384	95.90	1.00	93.84
*Dromaius novaehollandiae*	3317187	2777	97.50	0.40	98.59
*Homo sapiens*	30940000	2416	93.50	1.40	99.27
*Homo sapiens*	8420670	3103	92.80	1.40	99.53
*Mus caroli*	111200000	3162	93.50	1.80	99.60
*Falco peregrinus*	3918221	7020	97.30	0.30	99.68
*Equus asinus*	15030000	9021	95.60	0.60	99.76
*Phascolarctos cinereus*	11590000	1906	91.90	1.40	99.92
*Homo sapiens*	142200000	6505	92.00	1.20	99.97
*Camelus bactrianus*	8738935	35454	95.20	0.90	100.01
*Mus musculus*	6370149	9129	93.30	1.60	100.02
*Homo sapiens*	19360000	11138	93.20	1.40	100.03
*Homo sapiens*	5550336	10430	92.80	1.40	100.05
*Serinus canaria*	19890000	304399	96.50	0.40	100.05
*Desmodus rotundus*	26190000	29800	94.30	0.90	100.12
*Sarcophilus harrisii*	1770155	35974	89.60	1.40	100.53
*Homo sapiens*	195564	858918	86.10	1.30	100.97
*Gadus morhua*	258723	427427	60.60	0.90	107.48
*Ovis aries*	43840000	1217	38.10	0.30	115.73
*Homo sapiens*	16130000	43100	11.40	79.70	155.54
*Homo sapiens*	18070000	47409	11.10	80.00	155.86
*Acinonyx jubatus*	48410000	6438	8.70	85.90	160.17
*Salvelinus alpinus*	41440000	10	29.40	1.70	308.20
*Xiphophorus hellerii*	27850000	4	18.50	0.70	506.60

Duplicate species in the table represent various genome assemblies uploaded to NCBI

The N50 values alone might suggest that some of the *H. sapiens* assemblies are the best (N50s = 149,700,000 and 142,200,000), but even comparing these two N50s, the distances are 10.89 and 99.9 respectively. Upon investigation, the more distant assembly has 6,505 contigs, while the less distant assembly has only 24. The full name and descriptions of these assemblies is available in the GitHub repository.

## Conclusions

In conclusion, measures of contiguity are not reliable as the sole measure of genome assessment. Additional metrics can be added, but these measures may not be simple to obtain or may require a reference that does not exist for the species in question. An additional metric is presented that incorporates metrics obtained from publicly available tools and human subject matter expert knowledge and presents the user with a single score.

For researchers who are assembling new genomes, there are a variety of methods to ensure that their genome assembly is high quality. Tools like BUSCO and abyss-fac can give estimates of the contiguity and completeness of the assembly, similar to the statistics presented in this paper. Researchers can also use the estimated size of the genome as compared to the sum of the assembly as presented in abyss-fac. The quality of the assembly can also be checked with the reads used to make the assembly using CGAL and REAPR. REAPR can make corrections based on this information. A newly published tool, Referee [12], can use the per-base accuracy information in the reads to correct unlikely bases in the genome assembly.

Current work is underway to collect information from human subject matter experts about these assemblies in order to use machine learning to model the collective knowledge of experts. A website was developed that allows users to drag and drop genomes into a desired order and rank them from 1-10. These rankings can then be used to train a supervised machine learning algorithm and used to label unknown genomes based on metrics of the contiguity, completeness, and correctness.

## Abbreviations

BUSCOs: Benchmarking universal single-copy orthologs
NCBI: National center for biotechnology information
UCs: Ultraconserved elements
